# Concern About COVID‐19 Mediates the Relationship Between Life‐History Strategy and Stockpiling Food

**DOI:** 10.1002/ijop.70082

**Published:** 2025-07-11

**Authors:** Alyson Blanchard, Greg Keenan

**Affiliations:** ^1^ School of Health and Society University of Salford Salford Greater Manchester UK; ^2^ School of Psychology Liverpool John Moores University Liverpool UK

**Keywords:** children, COVID‐19, existential threat, life history theory, mortality salience, stockpiling food

## Abstract

Life‐history theory (LHT) charts the relationship of environmental conditions to resource allocation trade‐offs made by organisms to either reproduce or invest in somatic maintenance. Hazardous environments in which resources are unreliable should prompt adoption of a “fast” life‐history strategy in which short‐term gains are favoured. The COVID‐19 pandemic presents an opportunity to examine whether an increase in existential threat as signalled by a shift in environmental status impacted people's decision making in LHT‐relevant domains. In this online psychometric study (*N* = 274 individuals), we examined whether concerns about COVID‐19 mediated the relationship between life‐history strategy and the desire to have or have more children, and stockpiling food and household groceries. Contrasting results emerged. COVID‐19 concern mediated the relationship between LHS and stockpiling food and household groceries but not LHS and reproduction. These findings highlight potential differences in decision consequences or the type of shift in environmental conditions needed to prompt particular responses.

## 1 Introduction

Life history theory (LHT) provides a framework for understanding how environmental conditions shape an organism's life history trajectory—that is, their rate of maturation, reproductive age, and number of offspring (Hill 1993; Kaplan and Gangestad 2005). Due to the limited availability of finite resources, organisms have trade‐offs between allocating energy to reproducing sooner or later, which are shaped by the status of the environment. In dangerous environments where resources are unreliable, resource allocation should favour a fast life history strategy (LHS) of reproduction and more offspring. In comparison, safe and resource‐reliable environments should select a slow LHS of somatic investment and delayed reproduction in favour of fewer offspring (Pianka 1970). Either strategy is adaptive, conferring a fitness advantage in the corresponding environment.

LHT initially elucidated species‐level LHS differences in non‐human animals, although it has latterly been utilised in contextualising individual differences in human reproductive scheduling. For example, one particular focus concerns suboptimal childhood experiences such as stress, low‐quality parental attachment, and father absence, which are, as expected, connected to fast LHS reproductive scheduling markers such as faster maturation rate, increased number of sexual partners, and lower age at first child (Dunkel et al. 2015). From an evolutionary psychological perspective, personality and other dispositional traits should be co‐selected and arise such that they operate complementarily in facilitating an individual's LHS. This suite of traits has been latterly defined as Pace of Life Syndrome (POLS) (Nettle and Frankenhuis 2019), for which there is evidence. For example, slow LHS is associated with conscientiousness, anxiousness, and fearfulness, which are traits that indicate cautious interaction with the environment, as well as a future‐orientated perspective. In contrast, fast LHS is associated with low agreeableness and sensation seeking, which would expose an individual to environmental risk (Brüne 2016). Thus, people appear to think and behave teleologically with respect to their LHS. Furthermore, evolutionary fitness is afforded by the ability to respond to temporal and spatial shifts in the environment; therefore, even though an individual's LHS is established during childhood, it makes adaptive sense for their POLS to acclimatise to circumstances occurring in adulthood. Indeed, phenotypic plasticity is crucial to survival, as well as effective parenting; thus, it would be expected to endure throughout the life course (Trivers 1972). We would therefore expect POLS‐related behavioural changes in adults exposed to shifts in environmental conditions, especially when mortality salience is more prevalent. Indeed, behavioural plasticity acting upon childhood and adulthood experiences and current environmental conditions is demonstrated by increased risk‐taking and non‐delayed gratification behaviour in low childhood socio‐economic status individuals exposed to mortality salience and resource scarcity (Mittal and Griskevicius 2014). Like other animals, humans continually monitor the environment throughout the life course for condition‐contingent signals to adapt in ways that optimise survival and reproduction.

Even though previous research has examined the relationship between the quality of environment and reproductive scheduling (e.g., Dunkel et al. 2015), the current study seeks to utilise the unique set of circumstances presented by the COVID‐19 pandemic for probing phenotypic plasticity (i.e., behavioural changes) in the context of heightened existential threat in adult humans. In addition to operating within an environment which has rapidly changed due to the implementation of lockdowns, social distancing and facial coverings, people have been exposed to chronic and substantial media coverage about COVID‐19. Thus, mortality salience has potentially increased, especially in the initial emergence of the virus when it was novel as a concept and death rates were particularly high. Research has already demonstrated how individual differences that are proxies for LHS/POLS interplay with cognitive appraisals of and behavioural responses to COVID‐19. For example, individuals scoring higher in trait emotional intelligence, emotional stability, cognitive reserve as well as a positive approach to problem solving were able to engage in self‐regulated learning strategies more effectively in response to the stress and uncertainty caused by the pandemic (Albani et al. 2023). Being high in neuroticism had the reverse effect (Ikizer et al. 2022). Those with future‐oriented consciousness reported engagement with prevention measures and collective action, as well as compassion and concern for others (Lalot et al. 2021). In comparison, people who were impulsive and anxious were less likely to comply with prevention measures (Wismans et al. 2021). LHS/POLS proxies such as psychological distress, neuroticism, threat sensitivity and paranoia predicted stockpiling behaviour (Bentall et al. 2021). Furthermore, research investigating the COVID‐19 pandemic in relation to LHT specifically revealed that residents of Wuhan (regarded as the source for the outbreak of the virus) reported a faster LHS (Li and Cao 2021), while those with a slower LHS took precaution measures (Corpuz et al. 2020). Thus, the extant literature currently demonstrates the relationship between behaviours either directly or indirectly associated with LHS/POLS and the pandemic. The current study, however, seeks to build upon this by examining the impact of engagement in news about COVID‐19 on the acquisition of resources, namely stockpiling food and household groceries in addition to reproductive scheduling decisions. Such behaviour would be expected in response to an environment of increasing uncertainty and mortality salience. Specifically then, in this study, the influence of cognitive appraisals of the COVID‐19 pandemic and COVID‐19 news engagement will be investigated in relation to two LHS/POLS‐relevant criteria: the desire to have children or more children and stockpiling food and household groceries.

The following predictions are made:

1. A faster LHS/POLS will predict the desire to have children or want more children.
2. A slower LHS/POLS will predict increased food purchasing in response to the pandemic.
3. Slow LHS/POLS will predict increased concerns (i.e., the risk and consequences of catching) about COVID‐19.
4. In the framework of environmental signalling, increased COVID‐19 news engagement will positively predict increased concerns about COVID‐19.
5. Concerns about COVID‐19 will mediate the relationship between POLS and the desire to have more children and increased purchase of food and household groceries.

## 2 Method

### 2.1 Participants and Procedure

Two‐hundred and seventy‐four (Mean_age_ = 34.72, SD = 14.47, female = 202; male = 69; non‐binary [neither male nor female] = 1, prefer not to say [did not want to disclose] = 2) participants constituted a UK‐only (advertised within the UK only) convenience sample recruited via social networks and Call for Participants, an online platform used for the advertisement of studies to prospective participants (the recruitment parameters were set to the UK only). A £50 Amazon gift voucher was offered as an incentive to take part, and the winner was selected randomly from submitted email addresses. Data collection operated between April and May 2020.

The ethnic profile of the sample consisted of 80.7% White; 7.3% mixed race; 7.7% Asian; 2.2% Black; and 2.2% reported as “Other.” Participants were educated to bachelor's degree (39.4%), followed by A‐level/college (23.7%), master's (19%), GCSE (school) (8%), PhD (5.5%), and HND/BTEC/vocational equivalent level (4.4%). Similarly, 29.6% of participants were students, followed by 23% professional; 7.7% managerial; 6.9% customer service; 6.2% retired; 5.8% unemployed; 5.8% administrative; 4.4% associate professional; 4% skilled trade, and 1.5% temporary.

A brief description of the study was advertised with a link to the study, hosted on Qualtrics. On arriving at the landing page, participants were provided with information detailing the nature of the study and were asked to confirm they were over 18 years old and consented to taking part. Participants completed a series of self‐report psychometric measures and questions that took approximately 15 min to complete and were subsequently debriefed.

### 2.2 Measures

Unless otherwise stated, all measures utilised a 5‐point Likert scale (1 = strongly disagree, 2 = moderately disagree, 3 = neither disagree nor agree, 4 = moderately agree, 5 = strongly agree). For all measures, total scores were obtained by averaging across individual items' scores.

#### 2.2.1 LHS/POLS

The *K‐SF‐42 Short form of the Arizona Life History Battery* (Figueredo et al. 2017) was used, which taps into “behavioural and cognitive indicators of LH resource allocations among different domains of fitness” (Figueredo et al. 2017, 2). Items are scored using either a 7‐point (e.g., disagree strongly, agree strongly) or 4‐point (not at all, a lot) Likert scale. A total score was calculated for all items, with a higher score indicating a slower LHS. All of the following COVID‐19‐related variables were taken from Priniski and Holyoak (2022). *Perceived coronavirus severity* consisted of the following five items: “COVID‐19, commonly referred to as coronavirus, is no more severe than the flu”; “I am afraid of dying from or contracting coronavirus”; “Diseases that primarily affect the elderly are not that big of a deal”; “COVID‐19 is so rare there is no need for me to worry about it”; and “COVID‐19 is the biggest threat to public health in recent years.” A high score represented increased severity perception. *COVID‐19 prevention attitudes* were measured by six items including “It is important to protect others from COVID‐19”. Scores were reverse scored, so a higher score reflects increased prevention behaviours. *Intention to vaccinate* consisted of four items such as “A COVID‐19 vaccine will save lives.” A high score represents an increased intention to vaccinate. *Concerns about contracting COVID‐19* was measured via two items including “COVID‐19 is highly contagious and we must do what we can to prevent its spread.” A high score represents increased concern about contracting the virus. *Fear of COVID‐19* was measured using seven items that assesses participant's general fear of COVID‐19, including “I am most afraid of coronavirus‐19” and “I am afraid of losing my life because of coronavirus‐19.” A higher score reflects increased fear of COVID‐19. For *COVID‐19 news frequency*, participants used a 0–100 sliding scale (0 = infrequently, 100 = very frequently) to indicate how much COVID‐19‐related news they were engaging with.

#### 2.2.2 Stockpiling Food and Household Groceries

Participants were asked “In the last month, to what degree have you purchased more food and household groceries than you would usually?” Response options were: ‘has remained the same’, ‘slightly more’, ‘moderately more’, and ‘considerably more’. A high score represents increased food and household groceries purchasing.

#### 2.2.3 Desire to Have Children/More Children

Participants were asked “If you have children, how many more children would you like to have?” and “If you don't have children, how many children would you like to have?”

#### 2.2.4 Subjective Socio‐Economic Status

A 10‐point scale was used, with participants asked to indicate where they saw themselves on a scale of social standing relative to others, with a score of 1 being low and the top score of 10 being high.

#### 2.2.5 Education Level

Participants were asked to record their education level on a 7‐point scale, with GCSE/high school or equivalent as the lowest score and postgraduate as the highest.

#### 2.2.6 Demographics

Participants also reported their age, gender, and ethnicity.

### 2.3 Analytical Strategy

A structural equation model (SEM) was created to test whether concerns about the pandemic mediated the relationship between LHS scores and both stockpiling behaviour and desire for number of kids. All modelling was conducted in AMOS software version 28 (IBM, New York).

Data for two participants were removed as they provided missing data for key variables in the model, with complete datasets needed to calculate bootstrapped indirect effects. This left 272 usable responses.

To test model fit, a range of indices were generated. Standardised root mean residual (SRMR) values under 0.08 were considered indicative of good fit. A root mean square error of approximation (RMSEA) parsimony adjusted measure with values less than 0.06 is considered a good fit and values greater than 0.06 but less than 0.08 are considered acceptable (Hu and Bentler 1999). The Tucker–Lewis index (TLI) and comparative fit index (CFI) were deemed acceptable above 0.90 and good above 0.95 (Hu and Bentler 1999).

As several measures of concerns about COVID were collected, a separate confirmatory factor analysis (Bollen 1989) was completed prior to building the final model. A Maximum Likelihood Estimator was used with the same indices of model fit applied as above for the structural model.

The potential impact of both socioeconomic status markers (as measured via subjective social standing and education level) and gender on the two main outcome variables was tested via correlations and *t*‐tests respectively. In the interests of building a parsimonious structural equation model (Collier 2020), where these variables had no significant influence on the key outcome variables, they were omitted from the final model. However, the analyses with indicators of SES and gender are available on request.

## 3 Results

Descriptive statistics and correlations are reported in Table 1.

**TABLE 1:** Sample means (*M*), standard deviations (SD), Cronbach's alphas (*α*) and correlation coefficients.

	*M* (SD)	*α*	2	3	4	5	6	7	8	9	10	11	12
1. Life history strategy	99.37 (20.64)	0.87	0.12*	0.09	0.21***	0.09	0.12*	0.07	0.16**	0.03	0.09	0.01	0.12
2. COVID‐19 Contagion concern	1.76 (0.81)	0.68		0.63***	0.68***	0.46***	0.31***	−0.15*	0.13*	0.14*	0.28***	0.08	0.08
3. COVID‐19 Prevention attitudes	1.57 (0.57)	0.70			0.59***	0.33***	0.61***	−0.20***	−0.13*	0.20***	0.21***	0.02	0.11
4. COVID‐19 Preventing spread	2.09 (0.73)	0.80				0.40***	0.25***	−0.09	−0.056	0.13*	0.18**	0.10	0.01
5. COVID‐19 Intention to vaccinate	1.79 (0.83)	0.84					0.16**	−0.11	0.01	0.08	0.04	0.04	−0.03
6. COVID‐19 Fear	2.34 (0.86)	0.86						−0.16**	0.06	0.20***	−0.01	−0.01	0.04
7. COVID‐19 News frequency	4.9 (1.45)	NA							0.12	−0.13*	−0.26***	−0.18**	0.07
8. Desire to have/more children	2.90 (1.21)	NA								0.01	−0.42***	0.06	−0.06
9. Buying more food and groceries than usual	2.03 (1.05)	NA									−0.01	0.08	0.07
10. Age	34.7 (14.43)	NA										0.07	0.16**
11. Education	3.73 (1.22)	NA											0.09
12. Subjective social standing	5.32 (1.85)	NA											

*Note*: The numbers designating column headers correspond to the numbered variable names in the first column.

* *p* < 0.05.

** *p* < 0.01.

*** *p* < 0.001.

### 3.1 Latent Variable for Concern About COVID‐19

Several different measures relevant to concerns about COVID‐19 were collected (*Concerns about contracting COVID‐19*; *Perceived severity of COVID‐19*; *Prevention spread behaviour*; *Fear of COVID‐19*; *Intention to vaccinate*). To establish if these might load on to a latent variable for concerns regarding the pandemic, a confirmatory factor analysis was completed. Fear of COVID‐19 had a surprisingly weak loading on the concerns for COVID‐19 latent variable (*b* = 0.373), so it was removed from further analysis. The model was a good fit for the data (CFI = 0.999, TLI = 0.998, RMSEA = 0.022, SRMR = 0.015).

### 3.2 Structural Equation Model

**FIGURE 1:**
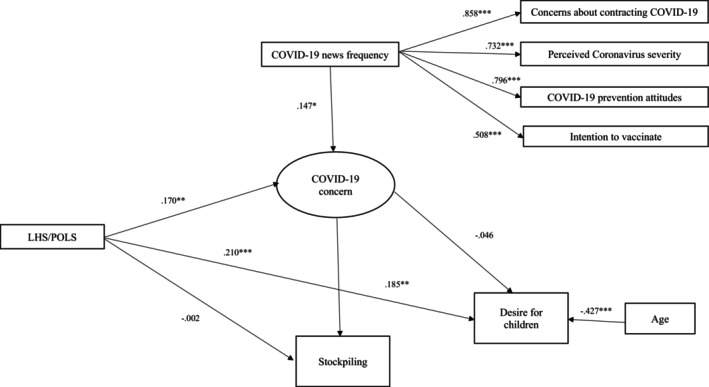
Associations between Life History Strategy (LHS)/Pace of Life Syndrome (POLS), concerns about COVID‐19, desire for having children and buying additional food and household groceries. Values are standardised regression coefficients *p < 0.05, **p < 0.01, ***p < 0.001. For ease of interpretation, residuals and covariances are not visually represented.

The final model was a good fit for the data (CFI = 0.953, TLI = 932, SRMR = 0.073, RMSEA =0.059). Direct associations between the variables and hypothesised indirect effects are shown in Tables 2 and 3, respectively. For ease of interpretation, the values in Figure 1 are standardised (*β*) coefficients, whereas those in Table 2 are unstandardised regression coefficients.

**TABLE 2:** Direct associations between variables (unstandardised regression coefficients).

Association	*b* (SE)	*p*	95% CI	*f* ^2^
LHS—Concerns about COVID‐19	0.011 (0.004)	0.008	0.005 to 0.019	0.03
COVID‐19 News Frequency—Concerns about COVID‐19	0.008 (0.003)	0.022	0.001 to 0.015	0.02
LHS—Buying more food and household groceries	0.000 (0.003)	0.969	−0.005 to 0.006	0.00
LHS—Desire for having more children	0.012 (0.003)	< 0.001	0.006 to 0.017	0.27
Concerns about COVID‐19—Buying more food and household groceries	0.140 (0.050)	0.005	0.061 to 0.231	0.04
Concerns about COVID‐19—Desire for having more children	−0.041 (0.051)	0.430	−0.147 to 0.043	0.01
Age—Desire for having more children	−0.036 (0.004)	< 0.001	−0.042 to −0.028	0.22

**TABLE 3:** Hypothesised indirect effects.

Association	*b* (SE)	*p*	95% CI	*v*
LHS—Concerns about COVID‐19—Desire for having more children	−0.008 (0.012)	0.471	−0.035 to 0.008	< 0.001
LHS—Concerns about COVID‐19—Stockpiling	0.031 (0.016)	0.010	0.008 to 0.059	0.001

### 3.3 Desire for Having Children

Contrary to H_1_ (that a faster LHS would predict a desire to have more children), it was a slower LHS/POLS that directly predicted an increased desire for children (see Table 2). However, contrary to H_5_ (that concerns about COVID‐19 would mediate the relationship between POLS and the desire to have more children and increased food consumption) there was no indirect association between LHS/POLS and desire for having children (see Table 3). Therefore, despite environmental signalling about volatile environmental conditions, this did not appear to influence people's desires to have more or less children. As would be expected, those who were younger desired having children or wanting to have more children.

### 3.4 Recent Food and Household Groceries Purchasing Behaviour

As can be seen from Figure 1 and Table 2, a slower LHS/POLS did not directly predict buying more food and household groceries, which had been originally predicted as part of H_2_ (that a slower LHS/POLS would predict increased food purchasing in response to the pandemic). However, as hypothesised in H_5_ (that concerns about COVID‐19 would mediate the relationship between LHS/POLS and the desire to have more children and stockpiling food and household groceries) an indirect relationship (see Table 3) existed between a slower LHS/POLS and increased food and household groceries purchases via concerns about COVID‐19. As such, if an individual had a slower LHS/POLS and was concerned about the pandemic, they reported stockpiling food and household groceries, demonstrating a pathway through which LHS/POLS influenced this behaviour. The other anticipated direct effects within this pathway were significant, with a slower LHS/POLS directly predicting increased pandemic concern (Table 2). In turn, greater pandemic concern predicted increased purchasing of food and household groceries. As was predicted in H_4_, increased COVID‐19‐related news engagement predicted more concern about the virus.

## 4 Discussion

At the biological level, LHT describes how reproductive scheduling is up‐ or down‐shifted according to environmental conditions (Hill 1993; Kaplan and Gangestad 2005). Correspondingly, POLS denotes the suite of psychological characteristics that facilitate an individual's LHS (Nettle and Frankenhuis 2019). From an evolutionary perspective, it is also adaptive to exhibit behavioural plasticity in response to elevated mortality salience (Trivers 1972). The unique and abrupt change in circumstances arising from COVID‐19 in which people were subject to ubiquitous news coverage of the virus as well as behavioural change in the form of lockdowns, social distancing, wearing face masks, and increased hygiene practices have provided the opportunity to examine whether the pandemic bore consequences for POLS‐related domains. Previous research had already demonstrated relationships between LHS/POLS proxies and behaviour elicited by the pandemic (e.g., Li and Cao 2021; Corpuz et al. 2020), and the current study sought to broaden this examination further by considering how environmental signalling derived through engagement with COVID‐19 news also impacted on the stockpiling of food and household groceries, as well as reproductive decision making.

Contrary to prediction, the current study revealed that the pandemic did not change people's LHS/POLS in the key domain of wanting or wanting more children, although slower LHS/POLS did directly. Earlier studies have similarly demonstrated trends in the opposite direction to what LHT would predict with respect to slower LHS/POLS individuals engaging in resource‐demanding and high‐emission lifestyles and lower fertility rates being associated with high‐risk environments in the context of increased future orientation (Caudell and Quinlan 2016). Evidently, incongruent variation in LHS/POLS is possible. Furthermore, the fact that the average age of the study cohort was 34 years old may reflect postponed reproductive effort that is characteristic of slow LHS/POLS. It could also be the case that families of fast LHS/POLS participants were already maximised, without the desire for further children. This has interesting implications as it suggests an optimal window of time for reproductive effort—for example, where it coincides with a drop in fertility associated with aging that may not impact those with a slow LHS/POLS in the same way because of somatic investment and resource availability. Beyond a certain point, even with a fast LHS/POLS, individuals may not desire children because it is no longer an optimal strategy. Alternatively, the finding that COVID‐19 concern did not predict wanting nor wanting more children suggests that a shift in LHS/POLS might require considerably more severe environmental change such as living in a warzone. Such a pattern may be inferred from the post‐World War 2 “baby boomer generation” or increase in Ukraine marriages and pregnancy rates since the outbreak of the Russo‐Ukrainian war (Hyde 2022). Indeed, there is current debate with regards to how “environmental harshness” can be judged (Stearns and Rodrigues 2020) and the decision to have a child is highly complex due to the time and resource investment involved. Additionally, individuals may have decided against having children because of the economic instability caused by the pandemic, as seen previously in the decline and sudden uptake in birth rates during and after World War 2 except in those countries that experienced economic prosperity because of the war (e.g., Sweden and Switzerland) (Dublin 1945).

In contrast, and this time in line with predictions, slower LHS/POLS individuals purchased extra food and household groceries when they reported higher COVID‐19 concern, which suggests a behavioural reaction to the existential threat posed by the virus. These findings dovetail with previous research that has linked food purchasing and LHT in evidencing a relationship between obesity and a preference for calorie dense and filling foods in conditions of environmental harshness, as well as over‐purchasing in high‐income families (Bentall et al. 2021; Dittmann and Maner 2017; Laran and Salerno 2013). Choosing to purchase additional food might also indicate a more fluid response to environmental conditions, as it is less consequential than having a baby and therefore requires less thought, which potentially explains the difference in findings from the current study in which an association was observed for the stockpiling of food and household groceries but not the desire for having children. Furthermore, if slow LHS/POLS individuals engage in future planning, then it is congruent that increased uncertainty about the environment will elicit increased future‐proofing behaviour, which is reflected in that they also reported increased COVID‐19 concern. These findings are interesting because they suggest that potentially, those with a slower LHS/POLS are more sensitive to detrimental changes to environmental conditions. That is, there may be a ceiling effect for those with a fast LHS/POLS in that their LHS does not accelerate any further when faced with further uncertainty. As such, it would be useful to examine whether and how individuals respond to environmental conditions of increasing stability (i.e., resource reliability and safety) with respect to their LHS/POLS.

There are a number of limitations with the current study. Perhaps most notably, a longitudinal design would have been more effective in ascertaining behavioural change in response to changes in the environment. The current study also included an imbalance of male to female participants, thereby making it difficult to fully test the influence of gender on the pathways investigated. Furthermore, there is also ongoing debate about whether it is possible to psychometrically measure LHS (c.f., Richardson et al. 2021) as well as the application of LHT to interindividual variation in human beings (Zietsch and Sidari 2019) and therefore it is necessary to consider these current findings in the spirit of exploration and proof of concept. We have attempted to address some of these issues with respect to the inclusion of POLS rather than only LHS. Nevertheless, studies do produce consistent evidence in support of some of the tenets of LHT (e.g., Webster et al. 2014) and it provides an effective and valuable framework for understanding behavioural change in response to existential threat. Such understanding might be useful in developing and implementing effective interventions in similar situations, as the panic buying of food and provisions is consistently evidenced as responses to pandemics and other resource‐limiting situations (Sim et al. 2020). Future research would benefit from utilising alternative measures and approaches in cross‐referencing self‐report psychometric scores of LHS that could include risk‐taking behaviours, financial decision‐making, and other manifested cognitive and behavioural proxies of LHS. It could also be argued that the finding that individuals reporting a slower LHS/POLS engaged in stockpiling of food and household groceries could be theoretically misaligned with what LHT would usually predict—that in fact fast LHS/POLS would be those that do this. However, previous research has demonstrated that slow LHS/POLS is related to high‐resource acquisition and consumption parental‐investment behaviours that actually increase carbon emissions, thereby paradoxically destabilising the survivability of future generations (Caudell and Quinlan 2016). In addition, low fertility rates were associated with high‐risk environments. These findings demonstrate how LHS/POLS may operate counter to what would be expected; however, they also reflect the complexity of the relationship between behaviour and the environment. An alternative explanation, however, is that according to Risk Sensitivity Theory (Houston and McNamara 1999), rapid changes in the environment invoke prudent behaviours in individuals at the slower end of the LHS/POLS continuum. Evidently, further research is needed to replicate and examine further the nature of the relationships revealed in the current study.

## Ethics Statement

The study was given a favourable review by (anonymised for review) University Ethics Committee and adhered to the British Psychological Society's Code of Conduct (2018) which is informed by the 1964 Declaration of Helsinki.

## Consent

Informed consent was obtained from all individual adult participants included in the study.

## Conflicts of Interest

The authors declare no conflicts of interest.

## Data Availability

The data that support the findings of this study are available from the corresponding author upon reasonable request.
